# Associated Anomalies and Complications of Multicystic Dysplastic Kidney

**DOI:** 10.3390/pediatric14030044

**Published:** 2022-09-01

**Authors:** Matjaž Kopač, Robert Kordič

**Affiliations:** 1 Division of Pediatrics, Department of Nephrology, University Medical Centre Ljubljana, Bohoričeva 20, 1000 Ljubljana, Slovenia; 2 Division of Surgery, Department of Pediatric Surgery, University Medical Centre Ljubljana, Bohoričeva 20, 1000 Ljubljana, Slovenia

**Keywords:** multicystic dysplastic kidney, congenital genitourinary anomalies, cryptorchidism, prenatal ultrasound, spontaneous involution

## Abstract

Background: To assess multicystic dysplastic kidneys (MCDK) in children, their complications and associated congenital genitourinary anomalies. Methods: Children with unilateral MCDK, evaluated between 2012 and 2020, were analyzed. In this retrospective study, data were obtained from electronic and paper health care records. Results: There were 80 children included. Follow-up time was 8.0 +/− 5.2 years (mean +/− standard deviation). None of them had hypertension. In total, 43.8% of the children had associated congenital genitourinary anomalies, most commonly cryptorchidism and vesicoureteral reflux (VUR), and 6.3% of these children had chromosomopathy. All of them had normal kidney function except one child with dysplasia of the contralateral kidney. Urinalysis was normal in 90% of children. Extrarenal malformations occurred in 22.5% of them. We observed spontaneous involution of MCDK in 38.8% of children in the observed period. Nephrectomy was performed in 12.5% of children, at an average age of 2.0 years. Conclusions: Children with a unilateral MCDK have a very good prognosis if the contralateral kidney is normal. Associated congenital genitourinary anomalies are common. Cryptorchidism was found to be the most common associated anomaly among boys, which is unique for this study. Most of these children do not suffer from hypertension, kidney dysfunction or other complications.

## 1. Introduction

The multicystic dysplastic kidney (MCDK) is a developmental abnormality that occurs due to the failure of union of the ureteric bud with the renal mesenchyme resulting in a non-functioning kidney that is replaced by non-communicating cysts of different sizes with no renal cortex and an atretic ureter. Therefore, it can occur only unilaterally in a surviving infant. It is usually a sporadic condition with an incidence of 1 in 2000–4000 births [1]. It can present as a unilateral abdominal mass on routine neonatal examination, but it is usually detected during prenatal ultrasound (US) examination [1]. The cause of MCDK is unclear. There may be an underlying genetic predisposition, according to studies that analyzed coding exons of genes associated with congenital anomalies of the kidney and urinary tract (CAKUT) [2,3,4]. One of them reported MCDK was associated with mutations in the *CHD 1L*, ROBO2, HNF 1B and *SALL1* genes [3]. The authors of this study tried to reveal the natural progress, complications and associated congenital genitourinary anomalies in children with MCDK, to assess their clinical significance and compare the results with other studies in different populations.

## 2. Materials and Methods

In this retrospective study, the clinical features of children with unilateral MCDK, detected in clinical practice, were analyzed. They were evaluated at the University Medical Centre Ljubljana (Division of Pediatrics, Department of Nephrology), between 2012 and 2020. Data were obtained from electronic health care records and, additionally, from paper health care records from the hospital archive, obtained after formal request. The follow-up time and mean age of spontaneous involution of MCDK are presented as mean +/− standard deviation in years. The age of diagnosis of cryptorcidism by a pediatric urologist and age of its surgery repair are presented as mean age with range in years due to the small sample size of this subpopulation. The research was approved by an independent national review body (Slovenian Society of Nephrology).

## 3. Results

There were 80 children with unilateral MCDK. There were 50 boys (62.5%) and 30 girls (37.5%) among them. The left kidney was affected in 41 (51.3%) and the right kidney in 39 (48.7%) of these children. Follow-up time was 8.0 +/− 5.2 years (mean +/− standard deviation, SD). They were detected with prenatal US in 82.3% (in 65 out of 79 cases with available data) and in the first weeks of life in the rest of them, at the average age of 0.1 year. Most of them had a dynamic radionuclide renal scan later that confirmed the absent function of MCDK. All of them had follow-ups with US at regular intervals, usually once a year (and later, once every two years), depending on associated anomalies or complications, if present. None of them had hypertension, defined as blood pressure above the 95th percentile. In total, 35 (43.8%) children had associated congenital genitourinary anomalies, and the most common of them are presented in Table 1.

Cryptorchidism was found to be the most common associated anomaly among boys in this study, present in 12 boys with MCDK (24%). The mean age at formal diagnosis by a pediatric urologist was 2.3 years (ranging from 0.4 to 6 years). However, seven of them were diagnosed before the age of 2 years. Five of them had left-sided, three right-sided and four bilateral cryptorchidism. All of the boys with MCDK had ipsilateral cryptorchidism. All of them had undergone surgery (except one who is waiting for it), performed at the mean age of 3.7 years (ranging from 0.5 to 6.1 years). In one boy with bilateral cryptorchidism and right-sided MCDK, there was an absent vas deferens on the right side. In another boy, vas deferens was injured during surgery but immediately repaired afterwards. Inguinal hernia was found and repaired during cryptorchidism surgery in eight boys, left-sided in four, right-sided in three and bilateral in one of them.

Five (6.3%) of these children had chromosomopathy and two among them (2.5%) had the 22q11.2 deletion syndrome (22q11.2 DS)—DiGeorge syndrome. Eight children (10%) had a urinary tract infection (UTI), confirmed with urinoculture. All of them had normal kidney function except one child with dysplasia of the contralateral kidney (with glomerular filtration rate 68 mL/min/1.73 m^2^). Therefore, normal kidney function was present in 78 out of 79 patients (98.7%) with available data. Urinalysis was normal in 72 children (90%), while 4 (5%) had microhematuria, 2 (2.5%) had proteinuria and 1 child had both. Extrarenal malformations (heart defects, intestinal malformations…) occurred in 18 out of 80 children (22.5%). There were no cases of Wilms tumor or other malignancies in this cohort of patients. We observed spontaneous involution of MCDK in 31 children (38.8%) in observed period, with average age of involution at 4.1 +/− 3.6 years. Nephrectomy was performed in 10 children (12.5%). The rest of them were monitored with serial US, clinically and with other tests, as appropriate. Family history on CAKUT was present in 34.4% (in 22 out of 64 cases with available data).

Nephrectomy was performed at an average age of 2.0 years. The reasons for nephrectomy were: persisting or unusually big MCDK in four of them (in addition, one of those had ureterovesical junction stenosis and occasional cyanosis of lower extremities), UTI at 3 months in one of them, ipsilateral hydroureter in one of them and contralateral antireflux (grade 5) surgery with simultaneous ectopic pelvic MCDK (near the urinary bladder) and ipsilateral ureterocele removal in another one. The indication for nephrectomy of MCDK was not clearly defined in the patient’s health care record in three of them.

## 4. Discussion

In the present study, MCDK occurs more frequently in boys than in girls, which is in agreement with previous studies [1,5]. The reason for this is not yet known and could be a topic for future research.

The left kidney is more often affected according to some studies [6], but not in this study, where the left and right kidney were equally affected. Several genitourinary anomalies have been described that may be associated with MCDK, such as renal hypoplasia, vesicoureteral reflux (VUR), ureterocele, pyeloureteric stenosis or genital abnormalities [7,8,9,10]. VUR is the most common among them, occurring in 16–21% of contralateral kidneys of affected patients [7,9,11], a proportion that is similar to this study. Voiding cystourethrography (VCUG) was not routinely performed on all patients with MCDK in our study but in those with urinary tract infection or US signs, suggesting VUR, such as urinary tract dilatation.

A published study of about 100 reports of MCDK demonstrated involution or regression of 60% of MCDK kidneys within the first three years of life, no clinically significant increased risk of hypertension compared with the general population and very low risk of developing a Wilms tumor (less than 1 in 2000 cases) [12], which is similar to this study. In a prospective study from a regional registry of patients with MCDK, 10% of prenatally detected MCDK had involuted by the time of the first postnatal US. Long-term follow-up (mean 10.1 years) demonstrated complete involution of 47% of MCDK kidneys at a 5-year follow-up. Contralateral abnormalities were observed in 14% of patients, including VUR (11%) and ureteropelvic junction obstruction (UPJ, in 3%). At a 10-year follow-up, only 2 out of 94 patients had hypertension. There was no reported malignancy during the follow-up period. The median estimated glomerular filtration rate in these patients was 93 mL/min per 1.73 m^2^; however, 43% had an estimated glomerular filtration rate between 60 and 90 mL/min per 1.73 m^2^ [13], unlike in this study. Contralateral abnormalities included VUR (17%), UPJ obstruction (4%) and megaureter (2%). At a mean follow-up of 5.9 years, 10% of patients had reduced kidney function. Nephrectomy was performed in 19.8% [14], which is slightly more often than in the present study.

Cryptorchidism was found to be the most common associated anomaly among boys in the present study, which is unique for this population. All of them had undergone surgery (except one) at a mean age of 3.7 years. Several children had postponed surgery, mainly due to respiratory infections and the resultant increased risk for anesthesia. In one boy with bilateral cryptorchidism and right-sided MCDK, there was an absent vas deferens on the right side, which might tie the two findings together. Inguinal hernia was found and repaired during cryptorchidism surgery in two thirds of these children. To the best of our knowledge, no other study about MCDK revealed such a high proportion of patients with this anomaly. Cryptorchidism is defined as hidden or obscure testis (in Greek) that is not within the scrotum and does not descend spontaneously into the scrotum by four months of age (or corrected age for premature infants). It is usually used interchangeably with the term undescended testis. A cryptorchid testis may be atrophic or ectopic as opposed to truly undescended. A testis that is not in the scrotum on physical exam is either palpable elsewhere or nonpalpable. A testis outside the scrotum and palpable can be either retractile (normally descended testes that can be pulled into a suprascrotal position by the cremasteric reflex), incompletely descended (within the inguinal canal or just outside) or ectopic (in the perineal, femoral or suprapubic region). A nonpalpable testis is usually either in the abdomen or atrophic or absent [15,16]. Two of the children in this study (2.5%) had 22q11.2 DS, and both of them had cryptorchidism of the left testis, requiring surgery. According to the results of a recent study, patients with 22q11.2 DS have increased rates of both MCDK and undescended testes [17], as confirmed in our study. In addition, *CRKL1* was identified recently, located between LCR22C-LCR22D within the 22q11.2 deletion, as the driver of renal defects in these patients [17,18].

## 5. Conclusions

Children with a unilateral MCDK have a very good prognosis if the contralateral kidney is normal. It affects boys more commonly than girls. Most of them are detected prenatally. Associated congenital genitourinary anomalies are common. We found undescended testis in boys to be the most common among them, which is the novel finding in our study. Most of these children do not have a UTI, hypertension, kidney dysfunction or other complications.

## Figures and Tables

**Table 1:** Most common associated congenital genitourinary anomalies in children with multicystic dysplastic kidney (MCDK).

Associated Congenital Genitourinary Anomalies	Number of Children Affected (%)
cryptorchidism ^a^	12 (24.0%)
vesicoureteral reflux	13 (16.3%)
urinary tract dilatation (hydronephrosis and/or hydroureter)	8 (10%)
ectopia of MCDK	3 (3.8%)
ureteropelvic junction obstruction	2 (2.5%)
Inguinal hernia	8 (10%)
ureterocele	4 (5%)
OHVIRA syndrome (obstructed hemivagina with ipsilateral renal agenesis—after spontaneous involution of MCDK) with doubled uterus	1 (1.25%)

Legend: ^a^ this anomaly can affect only boys and, consequently, the proportion of boys affected is higher compared to the proportion of the entire cohort of children.

## Data Availability

Available from the corresponding author upon reasonable request.
